# Improving Motor Imagery-Based Brain-Computer Interface Performance Based on Sensory Stimulation Training: An Approach Focused on Poorly Performing Users

**DOI:** 10.3389/fnins.2021.732545

**Published:** 2021-11-05

**Authors:** Sangin Park, Jihyeon Ha, Da-Hye Kim, Laehyun Kim

**Affiliations:** ^1^Center for Bionics, Korea Institute of Science and Technology, Seoul, South Korea; ^2^Department of Biomedical Engineering, Hanyang University, Seoul, South Korea; ^3^Department of HY-KIST Bio-Convergence, Hanyang University, Seoul, South Korea

**Keywords:** motor imagery, brain-computer interface (BCI), sensory stimulation training (SST), somatosensory attentional orientation (SAO), poor performer

## Abstract

The motor imagery (MI)-based brain-computer interface (BCI) is an intuitive interface that provides control over computer applications directly from brain activity. However, it has shown poor performance compared to other BCI systems such as P300 and SSVEP BCI. Thus, this study aimed to improve MI-BCI performance by training participants in MI with the help of sensory inputs from tangible objects (i.e., hard and rough balls), with a focus on poorly performing users. The proposed method is a hybrid of training and imagery, combining motor execution and somatosensory sensation from a ball-type stimulus. Fourteen healthy participants participated in the somatosensory-motor imagery (SMI) experiments (within-subject design) involving EEG data classification with a three-class system (signaling with left hand, right hand, or right foot). In the scenario of controlling a remote robot to move it to the target point, the participants performed MI when faced with a three-way intersection. The SMI condition had a better classification performance than did the MI condition, achieving a 68.88% classification performance averaged over all participants, which was 6.59% larger than that in the MI condition (*p* < 0.05). In poor performers, the classification performance in SMI was 10.73% larger than in the MI condition (62.18% vs. 51.45%). However, good performers showed a slight performance decrement (0.86%) in the SMI condition compared to the MI condition (80.93% vs. 81.79%). Combining the brain signals from the motor and somatosensory cortex, the proposed hybrid MI-BCI system demonstrated improved classification performance, this phenomenon was predominant in poor performers (eight out of nine subjects). Hybrid MI-BCI systems may significantly contribute to reducing the proportion of BCI-inefficiency users and closing the performance gap with other BCI systems.

## Introduction

A brain-computer interface (BCI) enables communication and control over computer applications and external devices directly from brain activity (Yao et al., 2017), and can improve the quality of life and independence of people with motor disabilities (Joadder and Rahman, 2017). Motor imagery (MI) can provide an intuitive mapping of direction between BCI interfaces and control commands better than other existing systems (i.e., steady-state visually evoked potential– and event-related potential–based BCI systems) because the required MI tasks would be closely associated with commands to control the external device (Batula et al., 2014). However, owing to the poor MI-BCI performance achieved thus far, this technique is a long way from providing interfaces that interact with external devices in applications in daily life. The performances of BCI systems feature a significant user-dependent difference; users can be categorized as either “good” or “poor.” Thus, one of the critical issues in MI-BCI studies is to improve performance and narrow the gap between good and poor performers. In addition, some users do not reach a sufficient level of accuracy (less than 70%) when trying to control an external device using a BCI system, a phenomenon called “BCI illiteracy” (Allison and Neuper, 2010; Yao et al., 2018) or “BCI inefficiency” (Shu et al., 2018; Zhang et al., 2019a). Studies revealed that 15–30% of users have BCI inefficiency and are unable to generate the proper brain rhythms even after BCI training (Leeuwis et al., 2021). Previous studies found that 55.6% (Lee et al., 2019) and 42.9% (Meng and He, 2019) of BCI-naïve participants were unable to achieve a 70% performance across one and three training sessions, respectively. BCI inefficiency is a significant problem that warrants research effort if these systems are to be useful in the future (Maskeliunas et al., 2016). Recent studies have tried to improve the classification performance of BCI inefficiency subjects using the deep learning method (i.e., convolutional neural network) because they cannot produce stronger contralateral ERD/ERS activity (Zhang et al., 2019b; Stieger et al., 2021; Tibrewal et al., 2021).

BCI research aimed at improving performance can focus either on technological factors (i.e., improvements in algorithms for feature extraction and classification) or on human factors (i.e., factors affecting how well a person generates quality EEG patterns). The latter has been relatively less studied (Penaloza et al., 2018), but many studies have attempted to improve the performance of MI-BCIs using a human-factors approach. This was done by assisting performance of the experimental task by providing visual feedback of the following kinds: (1) MI task results (i.e., direction, gauge bar) (Mousavi et al., 2017; Lukoyanov et al., 2018), (2) virtual reality environment (Skola et al., 2019; Choi et al., 2020), (3) neuro-feedback (Ono et al., 2018; Meekes et al., 2019), and (4) realistic visual feedback designed to induce a sense of embodiment (Alimardani et al., 2018) or a virtual-reality embodiment (Škola and Liarokapis, 2018). Other studies have demonstrated enhancement of MI-BCI performance resulting from improvements in training methods by use of haptics (Wu et al., 2017; Grigorev et al., 2021) and electric-stimulation feedback (Bhattacharyya et al., 2019). Haptic feedback can affect the modulation of beta (Wu et al., 2017) and mu (Grigorev et al., 2021) rhythms in the left or right sensorimotor cortex during hand movement. Another study reported the use of auditory feedback to improve MI performance by modulating sensorimotor rhythms (McCreadie et al., 2013). Lastly, studies have shown improvements in MI performance through multimodal feedback, including haptic and visual feedback (Lukoyanov et al., 2018; Wang et al., 2019), electrical stimulation, and visual feedback (Bhattacharyya et al., 2019).

Many previous studies have made efforts to improve MI performance by using visual, haptic, electrical, and auditory feedback, and some have reported improvements (Ladda et al., 2021). However, training using sensory stimulation with tangible objects has not been considered. MI is a challenging technique that often requires high concentration and extended training from users (Royer et al., 2010), and a substantial percentage of participants do not achieve good accuracy even after training (Guger et al., 2003). In this context, a hybrid training approach that combines two or more training methods can potentially improve the poor performance of some users by improving the consistency of the MI features detected by the BCI (Ramos-Murguialday et al., 2012). We focused on revising the training method to improve performance in poorly performing users by combining MI and somatosensory attentional orientation (SAO). SAO and MI are cross-modal mental tasks independent of exogenous stimuli and have different neurophysiological origins, namely the somatosensory and motor cortices, respectively (Yao et al., 2017). We believe that hybrid training methods can improve the performance of users compared to conventional methods that use only signals recorded over the motor cortex. Thus, this study explored a new hybrid imagery method to improve MI performance that employed three MI classes: left hand, right hand, and right foot. This approach involved training in the use of the sensations from tangible objects (i.e., a hard and rough ball) and motor execution (ME), where participants were instructed to imagine them at the same time.

## Materials and Methods

### Participants

For this study, we recruited 14 healthy participants, of which 7 were female and all were right-handed. The mean age of the participants was 27.21 ± 3.88 year. All participants in this experiment had prior experience with the motor imagery paradigm. Each was paid 90,000 KRW. None had any family or medical history of central nervous system disorders. Participants were asked to sleep normally and abstain from alcohol, cigarettes, and caffeine for 12 h before the experiment. Written consent from all participants was obtained after informing them of the restrictions and requirements of the experiments. This study was approved by the Ethics Committee of the Korea Institute of Science Technology, Seoul, South Korea (approval number: 2020-021).

### Electroencephalogram Acquisition

Electroencephalogram (EEG) signals were recorded at a sampling rate of 2,048 Hz using a BioSemi ActiveTwo system (BioSemi BV, Amsterdam, Netherlands) with 64 channels mounted on an EEG electrode cap arranged in the international 10--20 montage. The ground and reference electrodes were replaced by the common-mode sense and driven right-leg electrodes, which are specific to BioSemi systems^1^. EEG signals were down sampled to 256 Hz for analysis and the common-average reference was used for offline analysis. An infinite impulse response filter (1–50 Hz) and a wavelet-based neural network were applied to the raw EEG signals to remove artifacts (Nguyen et al., 2012).

### Experimental Design

**FIGURE 1:**
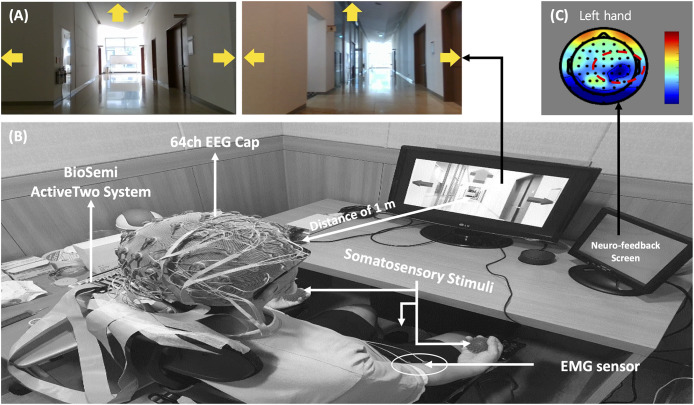
**(A)** Stimuli for motor imagery-based brain-computer interface task, **(B)** experimental environment, and **(C)** neuro-feedback mechanism.

This experiment utilized both MI (as control) and somatosensory-motor imagery (SMI) for BCI training. The participants sat in a comfortable armchair in an electrically shielded room. The monitor screen was placed 1 m from the participant, who was instructed to sit still, limit eye blinks, and minimize body, facial, and arm movements. The stimuli for MI were indicated using arrows on a three-way crossroads graphic (i.e., right, left, and forward). The stimuli were video clips presented before the crossroads was entered and located in the target direction. Participants then performed either the MI or the SMI task. The experimental environment and stimuli are presented in Figure 1.

**FIGURE 2:**
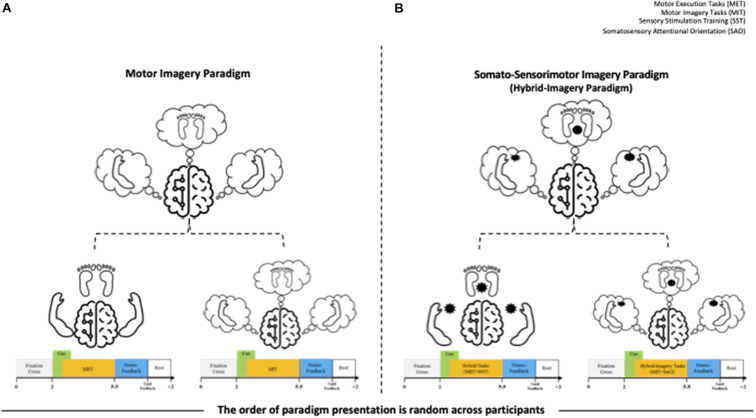
Overview of the experiments for the motor imagery (MI) and somatosensory-motor imagery (SMI) paradigms. **(A)** MI paradigm. The left hand signals MET_L_ and MIT_L_, the right hand signals MET_R_ and MIT_R_, and the right foot signals MET_F_ and MIT_F_. Subscripts: L, turn left; R, turn right; F, go forward. MET, motor execution task; MIT, motor imagery task. **(B)** SMI paradigm. The left hand signals SST-MET_L_ and SAO-MIT_L_, the right hand signals SST-MET_R_ and SAO-MIT_R_, and the right foot signals SST-MET_F_ and SAO-MIT_F_. SST, sensory stimulation training; SAO, somatosensory attentional orientation.

The MI paradigm consisted of a motor execution task (MET) and a motor imagery task (MIT). In the MET session, participants were required to perform movements of the left hand, right hand, or right foot, and the MIT session involved only imagining these physical movements (kinesthetic imagery). In the sessions, the movements of both hands and the right foot were cued by pictures of clenching hands and footprints, respectively. At the beginning of each trial, a “+” mark appeared in the center of the screen for 2 s. Then a cue arrow pointing to the left (MET_L_ and MIT_L_), right (MET_R_ and MIT_R_), or forward (MET_F_, MIT_F_) was presented visually on the three-way crossroad in the video clip. Participants were instructed to perform the task for 3 s after the cue appeared. After 0.5 s, neuro-feedback of the cortical activations around the motor and sensorimotor cortex (Figure 1) was presented until the participant responded with a keypress to indicate completion of the task. Each paradigm always started with the execution session (i.e., MET or SST–MET) and then proceed to the imagery session (i.e., MIT or SAO–MIT), as shown in Figure 2. In this study, neuro-feedback was based on EEG power in the frequency band 8–30 Hz recorded during the motor task. The sampling time window for power measurement was 2.4–4.4 s, which was the same length as that of the window for feature extraction for classification. After each run, we calculated the EEG power mapped to a cortical topography over one run and gave the subject feedback to increase motivation and to produce better results. Subjects could confirm the results of imagery task by the information of color and location in the brain topography, and it could have a positive effect on their task performance. The next trial started after subject’s feedback (Zoefel et al., 2011; Boe et al., 2014; Duan et al., 2021). To allow the subjects to concentrate on the experimental tasks, we designed the tasks to be administered when the subjects felt fully prepared. The procedures for MET and MIT for a single trial are shown in Figure 2A. Participants were required to perform a total of 150 MET trials (three classes: MET_L_, MET_R_, and MET_F_, 50 trials each) in three runs, and 300 MIT trials (three classes: MIT_L_, MIT_R_, and MIT_F_, 100 trials each) in six runs. Classes were presented in random order in each session.

**FIGURE 3:**
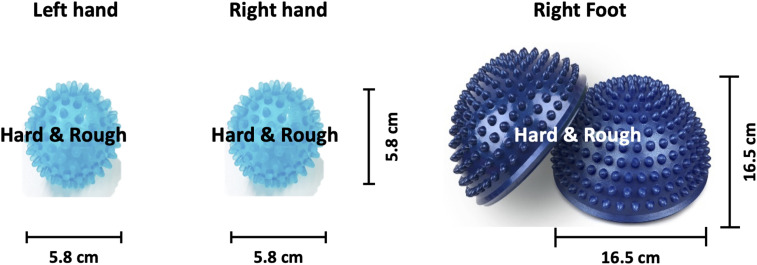
Image of the somatosensory stimulus. Hard and rough tangible balls were used as stimuli for the left and right hands while a hard and rough half-ball was the stimulus for the right foot.

The SMI paradigm consisted of sensory stimulation training (SST) with MET and SAO with MIT. In the SST–MET session, participants were required to clench hard and rough tangible balls in their left or right hands or step on a hard and rough half-ball, as shown in Figure 3. In the SAO–MIT session, participants were asked to use hybrid imagery for the movement of the left hand, right hand, and right foot that included the somatic sensory sensation of the tangible objects. Participants were required to perform a total of 150 SST–MET trials (three classes: SST–MET_L_, SST–MET_R_, and SST–MET_F_, 50 trials each) in three runs, and 300 SAO–MIT trials (three classes: SAO–MIT_L_, SAO–MIT_R_, and SAO–MIT_F_, 100 trials each) in six runs. As in MI tasks, the classes were presented in random order in each session. The procedure for a single trial was the same as in the MI paradigm (Figure 2B). This study used a “within subject” design and all participants were required to perform both MI and SMI paradigms. They experienced either the MI (i.e., MET/MIT) or the SMI (i.e., SST–MET/SAO–MIT) paradigm on the first day, and on the next day, they experienced the other paradigm at the same time of day (e.g., first day MI; second day, SMI; order randomized across participants). The SMI session was compared with the MI session to determine whether the classification performances of the three-class MI-BCI were improved between total, good, and poor performer groups. In addition, to improve the classification performance in the poor performer group, the cross-modality including eight combinations between MI and SMI were analyzed using two separate sessions with different trainings in mind. Some previous studies have reported that the proposed hybrid combination of SAO with MI has improved the classification performance in BCI-inefficient subjects (Yao et al., 2014, 2017). After each experiment paradigm, the subjects were required to report their feeling or experience for MI and SMI. The questions of the post-experimental interview are as follows. (1) Which experiment, MI or SMI, is more helpful to conduct imagery? (2) What makes MI or SMI so good? (open-end question).

**FIGURE 4:**
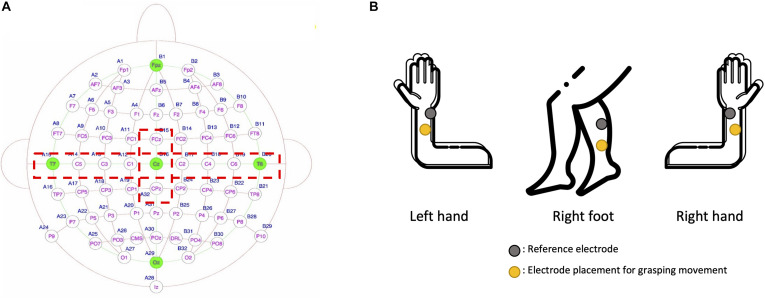
**(A)** Eleven electrode positions (red dotted lines) were used for electroencephalogram analysis. **(B)** Shown are electrode positions of the three channels in the electromyogram that were used to control by neuro-feedback the force and speed of movements (i.e., left hand, right hand, and right foot).

In this study, we recorded electromyogram (EMG) signals from the left hand, right hand, and right foot during the training sessions to rule out individual differences in force or speed. Previous studies have reported that the imagined force and speed of hand clenching affects brain oscillations (Fu et al., 2017; Xiong et al., 2019). The maximum voluntary contraction (MVC) of participants was measured, and they were asked to train at 50% of their MVC. Participant training was guided by feedback of force and speed results calculated from the EMG response and they were asked to maintain consistency in the imagination paradigm. The EMG feedback was presented as a sub-screen bar graph during the execution paradigm (i.e., in MET and SST–MET sessions). EMG electrodes were attached to the left hand, right hand, and right foot, as shown in Figure 4B. EMG signals were recorded at 2048 Hz using a BioSemi ActiveTwo system (BioSemi BV, Amsterdam, Netherlands) and six flat active electrodes. The high-pass and low-pass filters utilized were minimum order elliptic infinite impulse response type from MATLAB 2020a (MathWorks Inc., Natick, MA, United States). Raw EMG signals were down sampled to 256 Hz and the DC component removed using a 0.5-Hz high-pass filter. Finally, root mean square processing was performed and a 1-Hz low-pass filter was used for smoothing before calculation of the MVC.

### Feature Extraction

MI generates a spatial change in brain activity. To extract the spatial information from EEG signals, we used the Riemannian geometry approach with the spatial covariance matrices of each motor imagery task (Barachant et al., 2012). To obtain task-related spatial information, we extracted raw EEG data from the motor and somatosensory cortices through relevant EEG channels (i.e., FCz, C1, C3, C5, T7, Cz, C2, C4, C6, T8, and CPz; Figure 4A) followed by 8–30 Hz bandpass filtering. We selected the minimum number of EEG channels that can reflect the activation of both the motor and somatosensory cortices. The primary motor cortex and the somatosensory cortex are located anterior and posterior to the central sulcus, respectively, and the electrodes in the central area (i.e., C1, C2, C3, C4, C5, and C6) reflect the activation of both the motor and somatosensory cortex (Christensen et al., 2007; Yao et al., 2017). After the preprocessed EEG data were extracted from the time window of the motor imagery task (2.4–4.4 s), the epoched EEG data were transformed into symmetric positive-definite (SPD) matrices (Horev et al., 2016). We here calculated the SPD as a covariance matrix to extract the EEG spatial features for each motor imagery task. We denoted the pre-processed EEG signals as *X* ∈ *R*^*n* × *t*^ (*n* is the number of selected channels and *t* is the number of temporal data per trial). Based on the MI EEG signals, we estimated the normalized sample covariance matrix (SCM) as “***C***” in (1) (Ramoser et al., 2000).

(1) $$\text{C}=\frac{{\text{XX}}^{\text{T}}}{\text{trace}\,\left({\text{XX}}^{\text{T}}\right)},\left(\text{C}\in {\text{R}}^{\text{n}\times n}\right)$$

The SCM is an *n*×*n* SPD matrix with strictly positive eigenvalues *P*_*n*_ (*P*_*n*_ = {*PC*,*P* > 0}). The SPD matrices had dimensions *m* = *n* (*n* + 1)/2 because of their positive-definite and symmetric properties.

In this study, we used the median absolute deviation (MAD) to obtain the centrality characteristics of the EEG SPD matrices (Uehara et al., 2016). Using the MAD, the SPD matrices from the normalized covariance matrix for each motor task were arranged in a Riemannian manifold, which is a smooth manifold equipped with a high-dimensional Euclidean tangent space. Riemannian geometry is useful for identifying the brain information in EEG signals (Barachant et al., 2012). On the Riemannian manifold, we calculated the geodesic distance and mean of two SPD matrices with *P*_1_, …, *P*_*i*_, *P*_*j*_, …, *P*_*m*_, as shown below in (2)–(4):

(2) $${\text{P}}_{\mathrm{geodesic},i,\text{j}}=P_{\text{i}}^{\frac{1}{2}}{\left({\text{P}}_{\text{i}}^{-\frac{1}{2}}{\text{P}}_{\text{j}}{\text{P}}_{\text{i}}^{-\frac{1}{2}}\right)}^{\text{t}}{\text{P}}_{\text{i}}^{\frac{1}{2}},\left(\text{t}\in \left[0,1\right]\right)$$

(3) $${\text{P}}_{\text{distance},\text{i},\text{j}}={||\mathrm{Log}\,\left({\text{P}}_{\text{i}}^{-1}\,{\text{P}}_{\text{j}}\right)||}_{\text{F}}$$

(4) Pmean=∑i=1mP∈P⁢(n)argminPdistance2(P,Pi),(i,j∈m)

### Classification

After obtaining the centrality characteristics for each motor task using SPD matrices and the MAD strategy, we applied the Riemannian geometry-based classifier, which calculates the Fisher geodesic minimum distance to the mean (FgMDM) (Barachant et al., 2012). The FgMDM discriminates the classes of motor tasks by geodesic filtering. In this study, after we set the training covariance matrices (*C*_*train*_) and test covariance matrices (*C*_*test*_),*C*_*train*_ was projected into the tangent space. After the training data projection, we estimated the geodesic filters that maximize the between-class matrix and minimize the within-class matrix. The filtered features of each motor task in the tangent space were then projected onto the Riemannian manifold and were used to train the FgMDM classifier. Following this, *C*_*test*_ was also classified using the FgMDM after projection onto the geodesic filter of *C*_*train*_. We conducted this procedure with 10-fold cross-validation.

## Results

The average accuracy for all participants was 62.29 ± 16.96% for MI and 68.88 ± 14.04% for SMI. A paired *t*-test revealed a significant difference in the classification accuracy of MI and SMI (Shapiro-Wilk test, *p* = 0.560), with the performance of SMI better than that of MI (*t*_12_ = −2.669, *p* = 0.019, Cohen’s *d* = 0.423, with small effect size). Averaging over all participants, SMI achieved a 68.88% classification accuracy, 6.59% larger than that of MI, as shown in Table 1.

**TABLE 1:** Classification accuracy in the motor imagery (MI) and somatosensory-motor imagery (SMI) conditions.

*Class*	*S1*	*S2*	*S3*	*S4*	*S5*	*S6*	*S7*	*S8*	*S9*	*S10*	*S11*	*S12*	*S13*	*S14*	*Avg.*
*MI*	56.00	27.14	85.71	57.33	86.43	52.00	82.00	65.00	43.33	50.00	72.67	54.29	58.00	82.14	62.29
*SMI*	60.00	50.00	91.33	62.86	88.00	67.33	82.67	87.14	51.67	55.00	62.00	54.29	71.33	80.67	68.88
*SMI-MI*	4.00	22.86	5.62	5.52	1.57	15.33	0.67	22.14	8.33	5.00	–10.67	0.00	13.33	–1.48	6.59

*The bottom row presents the accuracy differences between conditions. S1–S14, all participants.*

**FIGURE 5:**
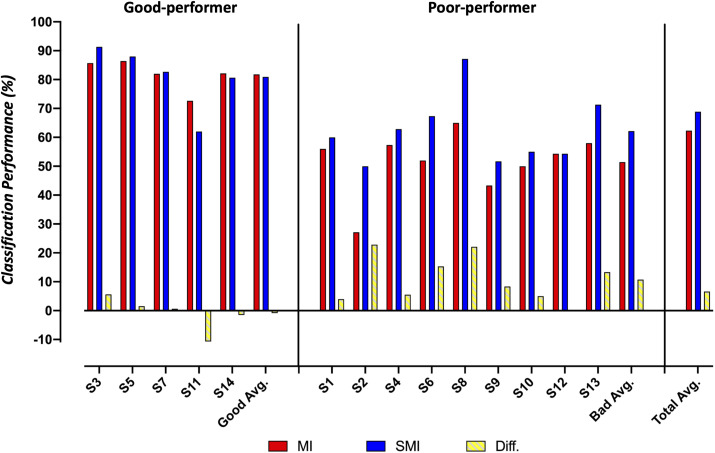
Comparison of the classification accuracy for motor imagery and somatosensory-motor imagery conditions between good and poor performer groups.

Figure 5 summarizes the classification performances of the good and poor performers in MI and SMI conditions. In our study, good performers were defined as those having more than 70% accuracy, a definition adopted from a previous study (Zhang et al., 2019a). In the poor performer group, the average classification performance on the SMI task was 10.73% larger than that on the MI task (62.18 ± 11.1% vs. 51.45 ± 10.27%). In contrast, the good performer group showed a slight reduction (0.86%) in performance on the SMI task compared to the MI task (80.93 ± 10.19% vs. 81.79 ± 4.91%).

**FIGURE 6:**
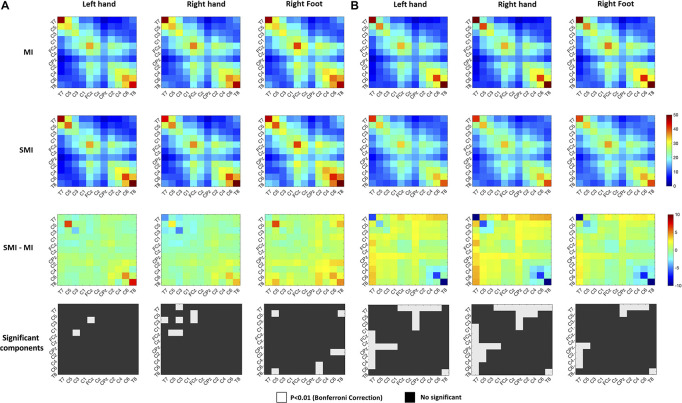
Comparison of the right-hand covariance matrix heat maps of the motor imagery and somatosensory-motor imagery conditions to show relationships among 11 selected channels. **(A)** Good performer group. **(B)** Poor performer group. Color scales show the paired t-statistic. The significance test was two-tailed.

We used heat maps to plot the covariance matrix pattern in MI and SMI conditions to observe the relationship among the 11 channels. For this, we conducted repeated paired *t*-tests on the components of the covariance matrix, using a Bonferroni-corrected *p*-value of 0.00008 (*p* = 0.01/121). For good performers, we isolated nine significant differences in the covariance matrix pattern between MI and SMI conditions. For poor performers, however, we found 30 significant differences in the connectivity among channels in the covariance matrices between MI and SMI conditions, as shown in Figure 6.

Table 2 shows the cross-modality classification results, where all eight (2^3) possible combinations for the two different modalities (MI and SMI) and for the three classes are presented. All combinations for each left hand, right hand, and right foot are as follows: MMM, SSS, SMM, SSM, SMS, MSM, MMS, and MSS. M and S mean motor image and somatosensory motor image, respectively, and the abbreviations presented in each condition are mapped in the order of the left hand, right hand, and right foot. For example, MSM condition is defined as a three-class analysis, with MI, SMI, and MI corresponding to left hand, right hand, and right foot, respectively. Classification performance of each pair of imagery tasks for all participants was 69.37–78.13% [72.13 ± 9.71% (SMM), 70.41 ± 7.85% (SSM), 78.13 ± 10.48% (SMS), 74.62 ± 10.87% (MSM), 69.37 ± 9.78% (MMS), and 75.62 ± 11.35% (MSS)], with SMS cross-modality achieving the best performance. The performance of the SMS condition was, on average, 15.84 and 9.25% greater than that of the MMM and SSS conditions, respectively. In the good performer group, classification performance of each pair of imagery tasks was 76.67–86.98% [83.23 ± 6.10% (SMM), 76.67 ± 6.04% (SSM), 86.98 ± 6.70% (SMS), 85.84 ± 5.49% (MSM), 79.90 ± 3.34% (MMS), and 86.05 ± 9.71% (MSS)]. The SMS condition achieved the best performance, which was, on average, 5.19 and 6.05% greater than in the MMM and SSS conditions, respectively. In the poor performer group, classification performance of each pair of imagery tasks was 63.52–73.22% [66.89 ± 5.54% (SMM), 66.93 ± 6.46% (SSM), 73.22 ± 8.85% (SMS), 68.39 ± 7.63% (MSM), 63.52 ± 6.84% (MMS), and 69.83 ± 7.35% (MSS)]. The SMS condition achieved the best performance, which was, on average, 21.76 and 11.04% greater than in the MMM and SSS conditions, respectively. Performances after optimized selection, which isolates the individual highest performance across cross-modalities, was on average 1.73, 2.23, and 1.45% higher in all participants, the good performer group, and the poor performer group, respectively.

**TABLE 2:** Classification accuracy of imagery tasks under cross-modality conditions.

**Participant**	**Imagery task**							
	**MMM**	**SSS**	**SMM**	**SSM**	**SMS**	**MSM**	**MMS**	**MSS**
***S1***	56.00	60.00	69.33	66.88	71.25	***71.25***	65.00	60.59
***S2***	27.14	50.00	60.63	***67.33***	65.00	57.65	56.88	61.33
*S3*	85.71	91.33	84.29	86.00	***97.14***	94.67	80.71	94.00
***S4***	57.33	62.86	76.67	65.00	76.67	***79.29***	64.67	69.29
*S5*	86.43	88.00	90.67	75.33	90.00	87.86	77.86	***93.57***
***S6***	52.00	67.33	69.33	64.67	***81.33***	78.00	64.00	78.67
*S7*	82.00	82.67	80.00	80.00	***84.67***	80.00	82.00	84.00
***S8***	65.00	87.14	70.00	80.77	***91.43***	74.17	76.15	80.77
***S9***	43.33	51.67	58.33	55.83	63.33	57.50	53.33	***67.50***
***S10***	50.00	55.00	63.33	61.67	62.50	63.33	***65.00***	61.67
*S11*	72.67	62.00	73.33	68.00	76.67	***80.00***	74.67	68.00
***S12***	54.29	54.29	63.08	***71.54***	71.43	65.00	70.67	71.33
***S13***	58.00	71.33	71.33	68.67	76.00	69.33	56.00	***77.33***
*S14*	82.14	80.67	87.86	74.00	86.43	86.67	84.29	***90.67***

**Averages**								

*Total sample*	62.29	68.88	72.73	70.41	***78.13***	74.62	69.37	75.62
*Good performer*	81.79	80.93	83.23	76.67	***86.98***	85.84	79.90	86.05
*Poor performer*	51.45	62.18	66.89	66.93	***73.22***	68.39	63.52	69.83

*Each task is described by a three-character code, i.e., MMM, SSS, SMM, SSM, SMS, MSM, MMS, and MSS; M: motor imagery, S: somatosensory-motor imagery. The first, second, and third letters in the code denote the left hand, right hand, and right foot, respectively. The participant numbers of those previously classified as “poor performers” on a pure M task are in bold, and the best classification results between the cross-modality conditions are in bold and italicized.*

Table 3 shows the results of interviews reported by subjects after each experimental paradigm. To question 1, the four good performers answered that the MI task was more helpful. They answered that the MI was more familiar than SMI as they had done it previously, and SMI was more confusing than MI. On the other hand, the eight poor performers answered that the SMI task was more helpful. Among them, four subjects reasoned that SMI felt more comfortable while performing kinesthetic MI and other three answered that tangible objects helped enable consistent imagery. In addition, subjects 10 (poor performer) and 11 (good performer) reported no significant difference between the two paradigms.

**TABLE 3:** Results for a subjective interview related to feeling or experience between MI and SMI paradigms.

**Participant**	**Question 1: Which experiment, MI or SMI, is more helpful to conduct imagery?**	**Question 2: What makes MI or SMI so good?**
***S1***	SMI	I don’t know clearly
***S2***	SMI	SMI is more comfortable to perform kinesthetic MI
*S3*	MI	MI is more familiar than SMI
***S4***	SMI	Tangible object (SMI) helps to make consistent imagery
*S5*	MI	MI is more familiar than SMI
***S6***	SMI	SMI is more comfortable to perform kinesthetic MI
*S7*	MI	MI is more familiar than SMI
***S8***	SMI	Tangible object (SMI) helps to make consistent imagery
***S9***	SMI	SMI is more comfortable to perform kinesthetic MI
***S10***	Anything is fine	Anything is fine
*S11*	Anything is fine	Anything is fine
***S12***	SMI	Tangible object (SMI) helps to make consistent imagery
***S13***	SMI	SMI is more comfortable to perform kinesthetic MI
*S14*	MI	MI is more familiar than SMI

*The poor performers are in bold.*

## Discussion

This work proposes a unique approach to improving MI-BCI performance for three classes by using a tangible object-based training method to enhance MI. Approaches to improving BCI performance are divided into technological factors (i.e., improving the algorithm) and human factors (i.e., improving the EEG patterns of the users) (Penaloza et al., 2018). Technological factors may have reached their limit in enhancing performance. In addition, the MI-BCI approach may have limitations in generating quality EEG patterns from users (Blankertz et al., 2006; Tangermann et al., 2012). This work proposes a new hybrid method of imagery that combines motor execution and somatosensory sensation from a tangible object to improve the MI performance of poorly performing users. The three classes showed an average accuracy in the SMI condition 6.59% larger than in the MI condition (68.88% vs. 62.29%, *p* < 0.05). In poor performers, SMI achieved an average classification performance 10.73% larger than did MI (62.18% vs. 51.45%). Good performers did not show a significant difference between the MI and SMI conditions. This work confirmed that methods using hybrid modalities (i.e., combining MI and SAO) can be useful alternatives to elementary modalities in improving the three-class BCI performance of poor performers.

The investigation of the effect of hybrid modality was motivated by how well a person can generate quality EEG patterns. First, this study attempted to achieve quality EEG patterns by enabling consistent MI using tangible objects. We confirmed that participants have different methods of MI for the task of imagining hand clenching. For instance, some imagined performing hand clenching (i.e., kinesthetic imagery) while others imagined watching a hand clenching (i.e., visual imagery). The participants performed tasks with their preferred method of MI and reported difficulties in using non-preferred methods. Inconsistent MI methods can adversely affect the quality of EEG patterns in participants (Neuper and Pfurtscheller, 2009; Wolpaw and Wolpaw, 2012). According to post-experiment interviews, participants in the poor-performer group who had experienced the hybrid modality were able to perform consistent MI by imagining the sensation of a tangible object (i.e., the imagination of hand clenching). This approach is considered to have a positive impact on generating quality EEG patterns as it enables participants to perform consistent MI. Furthermore, previous studies have reported significant differences in MI patterns depending on the force and speed of hand clenching (Fu et al., 2017; Geng and Li, 2020; Ortega et al., 2020). Here, we presented feedback to participants on the force and speed of hand and foot movements based on EMG signals (target: 50% of MVC) so that these parameters are stabilized across MI trials. If the force and movement speed are not controlled, the participant performs the MI task based on user characteristics. Movement was controlled in this study because it is a major factor that can affect the procedure for hypothesis testing.

Second, the SAO and MI modalities in hybrid BCI should enhance classification performance compared to that of the motor-cortex EEG only. SAO from the somatosensory cortex and MI from the motor cortex have distinctive neurophysiological origins (Yao et al., 2017). This study confirmed that both the somatosensory and motor cortices can be influenced by hybrid MI using tangible objects (i.e., a rough and hard ball), which leads to improved MI performance by generating quality EEG patterns. In the analysis of the feature covariance matrix, the SMI condition in the poor-performer group improved the connectivity among the 11 channels relative to the MI condition. These results support the idea that hybrid-modality imagery using tangible objects can generate quality EEG patterns. However, we found no significant changes in the good performer group. All participants underwent under the MI-BCI paradigm. The good-performer group was defined as participants who achieved high performance in the MI paradigm, and the poor performer group was defined as those who did not. According to the post-experiment interviews, the good performer group found the established MI method more familiar than SMI and found it difficult to imagine two modalities at once. The poor-performer group, however, found that SMI using a tangible object could help them to imagine more specifically and consistently than in the MI condition. In the classification results, the poor performer group showed better performance in the SMI condition than in the MI condition. The good performer group revealed no significant difference among the two paradigms. We thus believe that training methods to enable specific and consistent imagination can lead to the generation of quality EEG patterns and contribute to improving the performance of the poor performer who has previously reported difficulties with MI methods.

A limitation of this study is recruitment of only healthy subjects. MI-BCIs are commonly used in medical applications such as restoring motor functions in stroke patients. Because the stroke patients with loss of motor movement are unable to make voluntary movements, the established training process uses the passive movement (PM) by external devices instead of ME. A previous study reported that PM (i.e., the execution of a movement by an external agency without voluntary control) and MI induce similar EEG patterns over the motor cortex (Arvaneh et al., 2017). Many previous studies have tried using MI-based BCI control with passive motion for stroke patients, and significant positive results have been confirmed (Arvaneh et al., 2017; Cantillo-Negrete et al., 2018; Lu et al., 2020). Because a tangible object can be applied to a hand or foot when the patient performs a passive movement using an external agent, the proposed method may have a positive effect on stroke patients. This limitation requires further validation in future studies.

Previous research reported that a hybrid modality (MI and SAO with a vibration burst) achieved an average classification accuracy for classes 7.70% larger than did MI and 7.21% larger than did SAO (86.1% vs. 78.4% and 78.9%, respectively) (Yao et al., 2017). Another study showed that the hybrid modality group achieved an accuracy 11.13% higher than did the MI group and 10.45% higher than did the selective sensation group, using a vibration stimulus for the two classes (Yao et al., 2014). Moreover, prior studies have reported that hybrid modalities using somatosensory stimuli can improve MI-BCI performance. These studies applied MI and SAO stimuli to both hands (i.e., L-MI, R-SAO, R-MI, L-SAO). However, our study differs in that participants were instructed to simultaneously imagine MI and SAO stimuli on the hands and feet. Ours is an intuitive approach in which movements and sensations based on tangible objects (somatosensory signals) can be simultaneously imagined in the motor imagery procedure. The past studies did not employ intuitive imagining of two modalities simultaneously because vibration stimuli and motor actions are independent. However, the proposed method of this study allows intuitive imagining of two modalities because the motor action and the somatosensory stimulus (i.e., a tangible object) are dependent. For example, the users are able to imagine feeling the sense of a tangible object in the hand when their hand is grasping. The proposed hybrid imagery is consistent with the human mental model, and intuitive processes designed according to this mental model show high efficiency in behavior or action (Young, 2008). Our work demonstrated relatively good performance compared to previous studies, and can contribute to improving the performance of poorly performing users. This study has the limitation that the small sample size was too small to allow generalization of the results, requiring further validation in future studies with a larger sample.

This study compared performance in cross-modality conditions such as MMM, SSS, SMM, SSM, SMS, MSM, MMS, and MSS, involving mixtures of MI (“M”) and SMI (“S”) conditions on different body parts. (The order employed in the above three-letter codes is left hand, right hand, right foot.) In the poor performer group, the SMS cross-modality had the best performance, which was 21.76% larger than in MMM and 11.04% larger than in SSS (78.9%). The conditions with different modalities on the two hands (i.e., SMS, MSM, and MSS) had higher performance than did conditions with the same modality on the hands (i.e., MMM, SSS, SSM, and MMS). We believe that cross-modality combinations of MI and SMI help users to distinguish their hands better in imagination than do single modalities. A previous study reported that the cross-modalities of left-MI, right-SAO (84.1%), and right-MI and left-SAO (84.9%) showed a higher performance than having the same modality on the two hands, i.e., left-MI and right-MI (78.4%) and left-SAO and right-SAO (78.9%) (Yao et al., 2017). MI and SMI were conducted in two separate sessions with different trainings in mind. Thus, the results obtained from combining the experimental data of the two separate sessions may differ from the experimental results of a single session. The purpose of this study is to confirm the possibility of cross-modality, which will be verified through future research. Future studies are needed on cross-modality imagery using various somatosensory stimuli, such as tangible objects that feel rough and hard, rough and soft, or smooth and soft, or with vibration and electric stimuli.

In addition, this work placed the arrow on the sides of the display indicating the cue for the MI task. The arrows could be placed closer to the center of the screen rather than on the sides, to reduce eye movement and eye movement artifacts in the EEG. The neuro-feedback is also presented on the side screen, which can cause the EEG artifacts by eye movement. These issues need to be improved to minimize the effect of the artifacts in further study. In conclusion, this study proposes a unique hybrid-MI using a somatosensory stimulus to improve the MI-BCI performance of poorly performing users. The hybrid modality enabled consistent MI and activation of both the somatosensory and motor cortex using a rough and hard ball as the stimulus (SAO). This hybrid modality led to a significantly improved three-class MI-BCI performance in poor performers. In addition, we here confirmed that the cross-modality combination of MI and SMI performed better than did a single modality. However, only one type of somatosensory stimulus was assessed in this study. We believe that combinations of the different types of somatosensory stimuli alluded to above may significantly improve BCI performance and should be further investigated in future. The hybrid modality proposed in this study can help poor performers improve MI-BCI performance and BCI literacy, which can contribute to the practical use and uptake of BCI.

## Data Availability Statement

The raw data supporting the conclusions of this article will be made available by the authors, without undue reservation.

## Ethics Statement

This study was approved by the Ethics Committee of the Korea Institute of Science Technology in Seoul, South Korea (Approval No. 2020-021). The patients/participants provided their written informed consent to participate in this study.

## Author Contributions

SP: conceptualization, methodology, the experiment, and writing—original draft. JH: investigation, visualization, data analysis, and the experiment. D-HK: data analysis, data curation, and the experiment. LK: conceptualization, writing—review, and editing, and supervision. All authors contributed to the article and approved the submitted version.

## Conflict of Interest

The authors declare that the research was conducted in the absence of any commercial or financial relationships that could be construed as a potential conflict of interest.

## Publisher’s Note

All claims expressed in this article are solely those of the authors and do not necessarily represent those of their affiliated organizations, or those of the publisher, the editors and the reviewers. Any product that may be evaluated in this article, or claim that may be made by its manufacturer, is not guaranteed or endorsed by the publisher.

## Footnotes

1
http://www.biosemi.com/faq/cms&drl.htm

## References

[B1] Alimardani M. Nishio S. Ishiguro H. (2018). “Brain-computer interface and motor imagery training: the role of visual feedback and embodiment,” in *Evolving BCI Therapy-Engaging Brain State Dynamics*, Vol. 2 ed. Larrivee D. (London: IntechOpen) 64. 10.1371/journal.pone.0161945

[B2] Allison B. Z. Neuper C. (2010). “Could anyone use a BCI?,” in *Brain-computer interfaces*, eds Tan D. Nijholt A. (London: Springer), 35–54. 10.1007/978-1-84996-272-8_3

[B3] Arvaneh M. Guan C. Ang K. K. Ward T. E. Chua K. S. G. Kuah C. W. K. (2017). Facilitating motor imagery-based brain-computer interface for stroke patients using passive movement. *Neural. Comput. Appl.* 28 3259–3272. 10.1007/s00521-016-2234-7 29051688 PMC5626804

[B4] Barachant A. Bonnet S. Congedo M. Jutten C. (2012). Multiclass brain-computer interface classification by Riemannian geometry. *IEEE Trans. Biomed. Eng.* 59 920–928. 10.1109/tbme.2011.2172210 22010143

[B5] Batula A. M. Ayaz H. Kim Y. E. (2014). “Evaluating a four-class motor-imagery-based optical brain-computer interface,” in *Proceedings of the 2014 36th Annual International Conference of the IEEE Engineering in Medicine and Biology Society* (Chicago, IL: IEEE), 2000–2003. 10.1109/EMBC.2014.6944007 25570375

[B6] Bhattacharyya S. Clerc M. Hayashibe M. (2019). Augmenting motor imagery learning for brain–computer interfacing using electrical stimulation as feedback. *IEEE Trans. Med. Robot. Bionics* 1 247–255.

[B7] Blankertz B. Muller K. R. Krusienski D. J. Schalk G. Wolpaw J. R. Schlogl A. (2006). The BCI competition. III: validating alternative approaches to actual BCI problems. *IEEE Trans. Neural. Syst. Rehabil. Eng.* 14 153–159. 10.1109/TNSRE.2006.875642 16792282

[B8] Boe S. Gionfriddo A. Kraeutner S. Tremblay A. Little G. Bardouille T. (2014). Laterality of brain activity during motor imagery is modulated by the provision of source level neurofeedback. *Neuroimage* 101 159–167. 10.1016/j.neuroimage.2014.06.066 24999037

[B9] Cantillo-Negrete J. Carino-Escobar R. I. Carrillo-Mora P. Elias-Vinas D. Gutierrez-Martinez J. (2018). Motor imagery-based brain-computer interface coupled to a robotic hand orthosis aimed for neurorehabilitation of stroke patients. *J. Healthc. Eng.* 2018:1624637. 10.1155/2018/1624637 29849992 PMC5903326

[B10] Choi J. W. Huh S. Jo S. (2020). Improving performance in motor imagery BCI-based control applications via virtually embodied feedback. *Comput. Biol. Med.* 127:104079. 10.1016/j.compbiomed.2020.104079 33126130

[B11] Christensen M. S. Lundbye-Jensen J. Geertsen S. S. Petersen T. H. Paulson O. B. Nielsen J. B. (2007). Premotor cortex modulates somatosensory cortex during voluntary movements without proprioceptive feedback. *Nat. Neurosci.* 10 417–419. 17369825 10.1038/nn1873

[B12] Duan X. Xie S. Xie X. Obermayer K. Cui Y. Wang Z. (2021). An online data visualization feedback protocol for motor imagery-based BCI training. *Front. Hum. Neurosci.* 15:625983. 10.3389/fnhum.2021.625983 34163337 PMC8215169

[B13] Fu Y. Xiong X. Jiang C. Xu B. Li Y. Li H. (2017). Imagined hand clenching force and speed modulate brain activity and are classified by NIRS combined with EEG. *IEEE Trans. Neural. Syst. Rehabil. Eng.* 25 1641–1652. 10.1109/TNSRE.2016.2627809 27849544

[B14] Geng X. Li Z. (2020). Decoding fNIRS based imagined movements associated with speed and force for a brain-computer interface. *Int. J. Model. Identif. Control* 34 359–365.

[B15] Grigorev N. Savosenkov A. Lukoyanov M. Udoratina A. Shusharina N. Kaplan A. (2021). A BCI-based vibrotactile neurofeedback training improves motor cortical excitability during motor imagery. *bioRxiv* [Preprint]. 10.1101/2021.02.28.433220 34343094

[B16] Guger C. Edlinger G. Harkam W. Niedermayer I. Pfurtscheller G. (2003). How many people are able to operate an EEG-based brain-computer interface (BCI)? *IEEE Trans. Neural. Syst. Rehabil. Eng.* 11 145–147. 10.1109/tnsre.2003.814481 12899258

[B17] Horev I. Yger F. Sugiyama M. (2016). “Geometry-aware principal component analysis for symmetric positive definite matrices,” in *Proceedings of the Asian Conference on Machine Learning: PMLR*, Hamilton, 1–16.

[B18] Joadder M. A. M. Rahman M. K. M. (2017). A review on the components of EEG-based motor imagery classification with quantitative comparison. *Appl. Theory Comput. Technol.* 2 1–15.

[B19] Ladda A. M. Lebon F. Lotze M. (2021). Using motor imagery practice for improving motor performance–a review. *Brain Cogn.* 150 105705. 10.1016/j.bandc.2021.105705 33652364

[B20] Lee M. H. Kwon O. Y. Kim Y. J. Kim H. K. Lee Y. E. Williamson J. (2019). EEG dataset and OpenBMI toolbox for three BCI paradigms: an investigation into BCI illiteracy. *Gigascience* 8:giz002. 10.1093/gigascience/giz002 30698704 PMC6501944

[B21] Leeuwis N. Paas A. Alimardani M. (2021). Vividness of visual imagery and personality impact motor-imagery brain computer interfaces. *Front. Hum. Neurosci.* 15:634748. 10.3389/fnhum.2021.634748 33889080 PMC8055841

[B22] Lu R. R. Zheng M. X. Li J. Gao T. H. Hua X. Y. Liu G. (2020). Motor imagery based brain-computer interface control of continuous passive motion for wrist extension recovery in chronic stroke patients. *Neurosci. Lett.* 718:134727. 10.1016/j.neulet.2019.134727 31887332

[B23] Lukoyanov M. V. Gordleeva S. Y. Pimashkin A. S. Grigor’ev N. A. Savosenkov A. V. Motailo A. (2018). The efficiency of the brain-computer interfaces based on motor imagery with tactile and visual feedback. *Hum. Physiol.* 44 280–288.

[B24] Maskeliunas R. Damasevicius R. Martisius I. Vasiljevas M. (2016). Consumer-grade EEG devices: are they usable for control tasks? *PeerJ* 4:e1746. 10.7717/peerj.1746 27014511 PMC4806709

[B25] McCreadie K. A. Coyle D. H. Prasad G. (2013). Sensorimotor learning with stereo auditory feedback for a brain-computer interface. *Med. Biol. Eng. Comput.* 51 285–293. 10.1007/s11517-012-0992-7 23197181

[B26] Meekes J. Debener S. Zich C. Bleichner M. G. Kranczioch C. (2019). Does fractional anisotropy predict motor imagery neurofeedback performance in healthy older adults? *Front. Hum. Neurosci.* 13:69. 10.3389/fnhum.2019.00069 30873015 PMC6403184

[B27] Meng J. He B. (2019). Exploring training effect in 42 human subjects using a non-invasive sensorimotor rhythm based online BCI. *Front. Hum. Neurosci.* 13:128. 10.3389/fnhum.2019.00128 31057380 PMC6481252

[B28] Mousavi M. Koerner A. S. Zhang Q. Noh E. De Sa V. R. (2017). Improving motor imagery BCI with user response to feedback. *Brain Comput. Interfaces* 4 74–86.

[B29] Neuper C. Pfurtscheller G. (2009). “Neurofeedback training for BCI control,” in *Brain-Computer Interfaces*, eds Graimann B. Allison B. Z. Pfurtscheller G. (Berlin: Springer), 65–78. 10.1007/978-3-642-02091-9_4

[B30] Nguyen H. A. T. Musson J. Li F. Wang W. Zhang G. F. Xu R. (2012). EOG artifact removal using a wavelet neural network. *Neurocomputing* 97 374–389. 10.1016/j.neucom.2012.04.016

[B31] Ono Y. Wada K. Kurata M. Seki N. (2018). Enhancement of motor-imagery ability via combined action observation and motor-imagery training with proprioceptive neurofeedback. *Neuropsychologia* 114 134–142. 10.1016/j.neuropsychologia.2018.04.016 29698736

[B32] Ortega P. Zhao T. Faisal A. A. (2020). HYGRIP: full-stack characterization of neurobehavioral signals (fNIRS, EEG, EMG, Force, and Breathing) during a bimanual grip force control task. *Front. Neurosci.* 14:919. 10.3389/fnins.2020.00919 33192238 PMC7649364

[B33] Penaloza C. I. Alimardani M. Nishio S. (2018). Android feedback-based training modulates sensorimotor rhythms during motor imagery. *IEEE Trans. Neural. Syst. Rehabil. Eng.* 26 666–674. 10.1109/TNSRE.2018.2792481 29522410

[B34] Ramoser H. Muller-Gerking J. Pfurtscheller G. (2000). Optimal spatial filtering of single trial EEG during imagined hand movement. *IEEE Trans. Rehabil. Eng.* 8 441–446. 10.1109/86.895946 11204034

[B35] Ramos-Murguialday A. Schurholz M. Caggiano V. Wildgruber M. Caria A. Hammer E. M. (2012). Proprioceptive feedback and brain computer interface (BCI) based neuroprostheses. *PLoS One* 7:e47048. 10.1371/journal.pone.0047048 23071707 PMC3465309

[B36] Royer A. S. Doud A. J. Rose M. L. He B. (2010). EEG control of a virtual helicopter in 3-dimensional space using intelligent control strategies. *IEEE Trans. Neural. Syst. Rehabil. Eng.* 18 581–589. 10.1109/TNSRE.2010.2077654 20876032 PMC3037732

[B37] Shu X. Chen S. Yao L. Sheng X. Zhang D. Jiang N. (2018). Fast recognition of BCI-inefficient users using physiological features from EEG signals: a screening study of stroke patients. *Front. Neurosci.* 12:93. 10.3389/fnins.2018.00093 29515363 PMC5826359

[B38] Škola F. Liarokapis F. (2018). Embodied VR environment facilitates motor imagery brain–computer interface training. *Comput. Graph.* 75 59–71.

[B39] Skola F. Tinkova S. Liarokapis F. (2019). Progressive training for motor imagery brain-computer interfaces using gamification and virtual reality embodiment. *Front. Hum. Neurosci.* 13:329. 10.3389/fnhum.2019.00329 31616269 PMC6775193

[B40] Stieger J. R. Engel S. A. Suma D. He B. (2021). Benefits of deep learning classification of continuous noninvasive brain-computer interface control. *J. Neural. Eng.* 18:046082. 10.1088/1741-2552/ac0584 34038873 PMC9305984

[B41] Tangermann M. Muller K. R. Aertsen A. Birbaumer N. Braun C. Brunner C. (2012). Review of the BCI competition IV. *Front. Neurosci.* 6:55. 10.3389/fnins.2012.00055 22811657 PMC3396284

[B42] Tibrewal N. Leeuwis N. Alimardani M. (2021). The promise of deep learning for BCIs: classification of motor imagery EEG using convolutional neural network. *bioRxiv* [Preprint]. 10.1101/2021.06.18.448960 PMC9307149 35867703

[B43] Uehara T. Tanaka T. Fiori S. (2016). “Robust averaging of covariance matrices by riemannian geometry for motor-imagery brain–computer interfacing,” in *Proceedings of the Advances in Cognitive Neurodynamics (V)* (Berlin: Springer), 347–353.

[B44] Wang Z. Zhou Y. Chen L. Gu B. Liu S. Xu M. (2019). A BCI based visual-haptic neurofeedback training improves cortical activations and classification performance during motor imagery. *J. Neural. Eng.* 16:066012. 10.1088/1741-2552/ab377d 31365911

[B45] Wolpaw J. Wolpaw E. W. (2012). *Brain-Computer Interfaces: Principles and Practice.* Oxford: Oxford University Press.

[B46] Wu H. Liang S. Hang W. L. Liu X. L. Wang Q. Choi K. S. (2017). Evaluation of motor training performance in 3D virtual environment via combining brain-computer interface and haptic feedback. *Adv. Inf. Commun. Technol.* 107 256–261. 10.1016/j.procs.2017.03.096

[B47] Xiong X. Fu Y. Chen J. Liu L. Zhang X. (2019). Single-trial recognition of imagined forces and speeds of hand clenching based on brain topography and brain network. *Brain Topogr.* 32 240–254. 10.1007/s10548-018-00696-3 30599076 PMC6373301

[B48] Yao L. Meng J. Zhang D. Sheng X. Zhu X. (2014). Combining motor imagery with selective sensation toward a hybrid-modality BCI. *IEEE Trans. Biomed. Eng.* 61 2304–2312. 10.1109/TBME.2013.2287245 24235291

[B49] Yao L. Mrachacz-Kersting N. Sheng X. Zhu X. Farina D. Jiang N. (2018). A multi-class BCI based on somatosensory imagery. *IEEE Trans. Neural. Syst. Rehabil. Eng.* 26 1508–1515. 10.1109/tnsre.2018.2848883 29994123

[B50] Yao L. Sheng X. Zhang D. Jiang N. Mrachacz-Kersting N. Zhu X. (2017). A stimulus-independent hybrid BCI based on motor imagery and somatosensory attentional orientation. *IEEE Trans. Neural. Syst. Rehabil. Eng.* 25 1674–1682. 10.1109/TNSRE.2017.2684084 28328506

[B51] Young I. (2008). *Mental Models: Aligning Design Strategy With Human Behavior.* New York, NY: Rosenfeld Media.

[B52] Zhang R. Li X. Wang Y. Liu B. Shi L. Chen M. (2019a). Using brain network features to increase the classification accuracy of MI-BCI inefficiency subject. *IEEE Access* 7 74490–74499.

[B53] Zhang R. Wang Y. Li X. Liu B. Zhang L. Chen M. (2019b). Deep learning of motor imagery EEG classification for brain-computer interface illiterate subject. *Annu. Int. Conf. IEEE Eng. Med. Biol. Soc.* 2019 3087–3090. 10.1109/EMBC.2019.8857923 31946540

[B54] Zoefel B. Huster R. J. Herrmann C. S. (2011). Neurofeedback training of the upper alpha frequency band in EEG improves cognitive performance. *Neuroimage* 54 1427–1431. 20850552 10.1016/j.neuroimage.2010.08.078

